# Prioritising patient involvement in patient reported outcome measures– a PROMising way to improve headache care

**DOI:** 10.1186/s10194-025-02019-x

**Published:** 2025-04-09

**Authors:** Lakshini Gunasekera, Jason C. Ray, Neha Kaul, Helmut Butzkueven, Elspeth Hutton, Terence J. O’Brien

**Affiliations:** 1 Department of Neurosciences, School of Translational Medicine, Monash University, Melbourne, Australia; 2 Department of Neurology, Alfred Health, Melbourne, Australia; 3 Department of Neurology, Austin Health, Melbourne, Australia; 4 Department of Nutrition and Dietetics, Alfred Health, Melbourne, Australia

**Keywords:** Headache, Migraine, Patient-reported outcome measures

## Abstract

**Background:**

The optimal management of migraine involves care strategies that reflect what matters most to patients. This usually involves an assessment of treatment efficacy with respect to headache reduction, safety of prescribed medications and overall patient satisfaction and/or improved quality of life. Traditionally, neurologists focus on objective measures such as monthly reductions to headache and migraine days from baseline. This is complemented with various patient reported outcome measures (PROMs) to quantify morbidity and treatment effect from the patient’s perspective. We present a review of currently available headache specific PROMs to summarise the design, key attributes, response format, recall period and length of questionnaires.

**Methods:**

A literature search was conducted using OVID Medline, Embase and Cochrane Library. The search strategy involved: (satisfaction OR patient satisfaction OR efficacy OR effectiveness) AND (disability OR morbidity OR burden OR severity OR impact OR patient reported outcomes OR PROMs OR outcome measures OR MIDAS OR HIT6 OR HDI OR MSQ OR MIG-SCOG OR Eq. 5D OR WPAI OR PGIC OR quality of life or QOL) AND (migraine OR chronic migraine OR headache OR primary headache OR cephalalgia OR headache disorder). A total of 16,024 articles returned. Removal of duplicates (*n* = 111), title and abstract screening (*n* = 15,853) and subsequent full text analysis (*n* = 19), left 41 articles. Reviewer comments led to addition of further 3 articles to our review. In total, of 44 included articles there were 20 headache-specific PROMs analysed.

**Results and conclusion:**

Our findings show that there is a significant lack of patient involvement in creation of headache PROMs thus there may be a gap between perceived treatment efficacy from the perspective of neurologists and that of patients. We suggest future assessment of migraine treatment efficacy considers what is important to the patient as a priority, in an effort to improve satisfaction with care.

## Introduction

“The patient with a headache often finds himself a medical orphan. He is fortunate indeed if his headache is transient, for otherwise he may find himself on an excursion to the opthalmologist [sic], otolaryngologist, neurologist, dentist, psychiatrist, chiropractor, and the latest health spa. He is x-rayed, fitted with glasses, analyzed, massaged, relieved of his turbinates and teeth and too often emerges with his headache intact.” [1].

The above quote by Packard [1], highlights the importance of communication between the patient and headache physician in order to ensure satisfaction for both parties. Physicians are taught to obtain a thorough medical history, conduct a comprehensive clinical examination, order relevant tests, refer to relevant specialists if required and prescribe medications to ideally cure, or manage bothersome symptoms. However, this path of action may not necessarily be what the patient is wanting or even expecting on presentation. How do we know if the above actions are done to please the patient with an answer and treatment, or is it merely to satisfy ourselves as doctors? Ideally there is no disconnect between the expectations of the doctor or the patient but all too often there is.

Migraine, as the most common neurological disease that places a burden on individuals and society at large, should be a public health priority in management [2, 3]. Adequate management requires input from the patient to ensure that our approach is in line with their priorities and wishes. If their perspective is not heard, valued and incorporated into our management strategies, then we risk under-treatment for the individual but also place further strains on finite public health resources.

Communication is important in ensuring that a therapeutic plan is created in line with the patient’s wishes. As migraine symptoms can be incredibly variable between patients, objective measures of treatment efficacy are valuable in tracking treatment progress over time. We know that patients can be variably affected with migraine with any or all of head pain, photophobia, phonophobia, dizziness, cognitive complaints, mood changes, disturbed sleep, productivity loss in social life or paid work, relationship struggles, a feeling of lack of control about one’s life due to unpredictable pain episodes, or fear of impending attacks [4]. Ideally we would want migraine and its treatments to have a positive impact on a patient’s quality of life, which is the ability of the patient to play their role in society to the utmost within the context of their culture, values systems, goals and concerns [4].

### The mismatch in expectations between patients and Doctors

Packard [1] conducted a survey of 100 patients presenting to a headache clinic, and 50 headache specialists. Interestingly, less than a third of patients reported pain relief as the main priority of their visit; nearly half reported wanting to know the cause of their headache as the main reason for presentation. There is a great deal of harm which can occur when communication is not optimised: it could reduce doctor/patient rapport and trust, lead to loss of crucial information vital for diagnosis, and lead to erroneous management pathways that are not in line with patient wishes.

A Korean study has replicated similar findings [5] to Packard [1]; a survey of 207 headache patients across 11 different clinics found that less than a third of patients were satisfied with the care offered by their headache specialist and that satisfaction was highest when patients were offered adequate explanations for their pain rather than chasing pain reduction. In a similar vein, an audit of patients attending a headache clinic in the United Kingdom found that 77% of patients wanted further explanations about what was causing their head pain; 20% of patients overall reported that they did not want symptomatic treatment of their headaches and were merely presenting for an explanation as to what was causing the pain in the first place [6]. This is an interesting finding because even if doctors offer symptomatic treatments to effectively reduce monthly headache days, which would be seen as ‘successful medical therapy’ from the doctor’s perspective, patients may leave feeling unsatisfied. From a patient perspective, it may be difficult to conceive that pain can be adequately treated without knowing the underlying cause while physicians may be satisfied with this approach because initial investigations have not revealed an underlying sinister cause. Bridging this gap in communication has the potential to greatly reassure patients and improve the relationship between the healthcare provider and patient.

In contrast, another study of differences between patient and physician expectations found that a patient’s main priority when attending a headache clinic was complete disappearance of pain, while the physician’s main priority was ensuring the safety of prescribed medications [7]. The likely reason for the disparity is probably the consequences– the patient with untreated pain will suffer at home, while the physician prescribing intolerable medications may lose rapport with the patient and may need to further manage side effects.

As the above studies show, there can be quite a stark contrast between why a patient attends a headache clinic and what the treating doctor assumes this reason to be based on their training and experience. Unless we can bridge this gap, patients may leave feeling unsatisfied despite the apparent ‘success’ in therapy that doctors cite based on their objective measurements.

### Search methodology

**Fig. 1:**
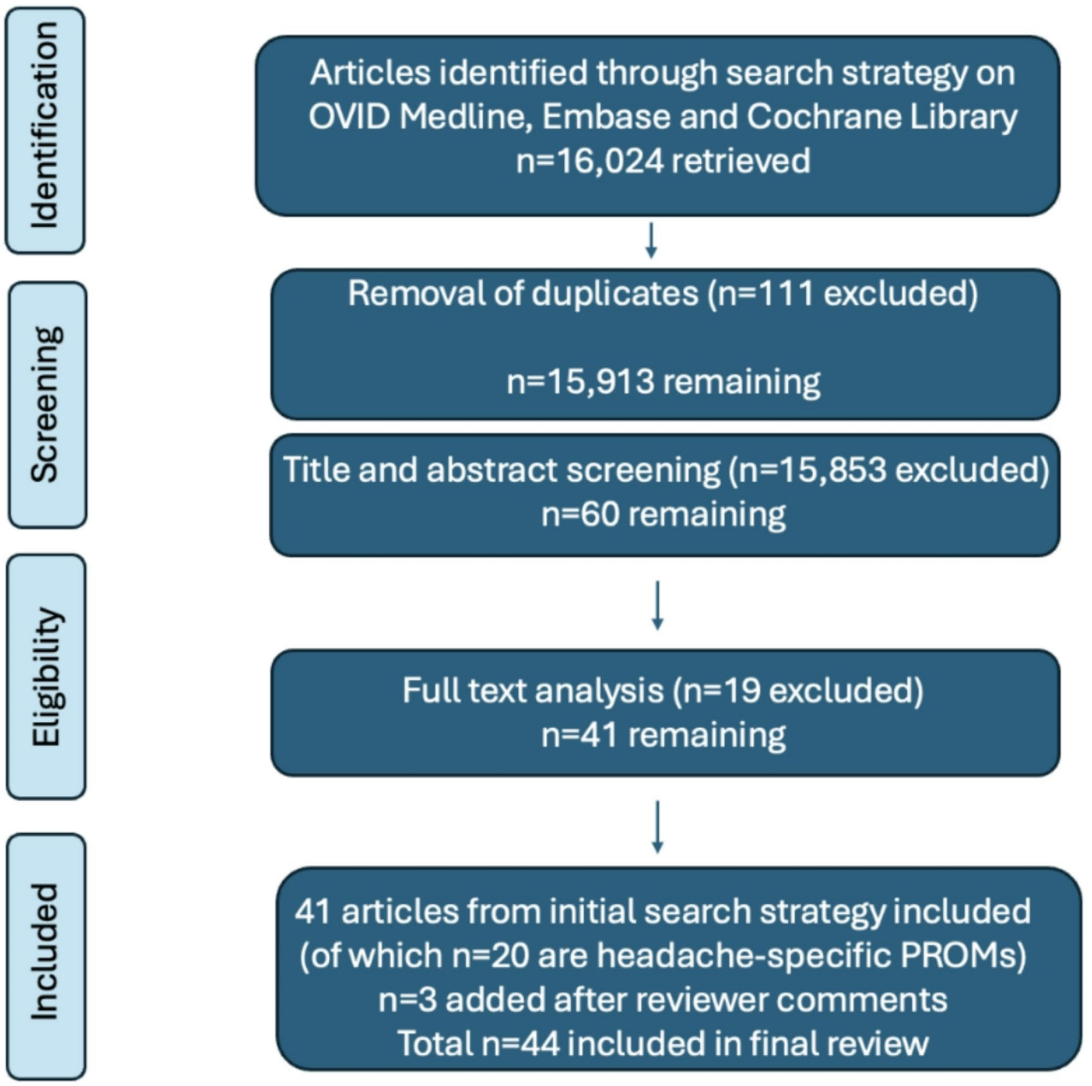
Graphical algorithm of search strategy and article selection

We aimed to summarise the currently available objective measures of headache management to check concordance between patient and physician expectations in migraine management. Our structured search strategy (without language or date restrictions) involved: (satisfaction OR patient satisfaction OR efficacy OR effectiveness) AND (disability OR morbidity OR burden OR severity OR impact OR patient reported outcomes OR PROMs OR outcome measures OR MIDAS OR HIT6 OR HDI OR MSQ OR MIG-SCOG OR Eq. 5D OR WPAI OR PGIC OR quality of life or QOL) AND (migraine OR chronic migraine OR headache OR primary headache OR cephalalgia OR headache disorder). This strategy was entered into three databases: OVID Medline, Embase and Cochrane Library. A total of 16,024 articles returned. Removal of duplicates (*n* = 111), title and abstract screening (*n* = 15,853) and subsequent full text analysis (*n* = 19), led to 41 articles included in this review. Of these 41 articles, 20 are migraine-specific PROMs which have been analysed. Reviewer comments led to inclusion of a further three references in our review. See Fig. 1 below for the search strategy represented as a graphical algorithm for the included headache PROMs.

The 20 PROMs included in our review are: Headache Disability Inventory (HDI), Headache-Specific Disability Questionnaire (HDQ), Headache Impact Test 6 (HIT6), Migraine Disability Assessment (MIDAS), Migraine-Specific Quality of Life version 2.1 (MSQv2.1), Migraine-Specific Quality of Life (MSQOL), Headache Needs Assessment (HANA), Eurolight, Completeness of response to migraine therapy (CORS), Migraine assessment of current therapy (Migraine-ACT), Migraine Therapy Assessment Questionnaire (MTAQ), Migraine Treatment Optimisation original/ 5-questionnaire/15-questionnaire (M-TOQ, M-TOQ-5 and M-TOQ-15), Patient Perception of migraine treatment revised (PPMQ-R), Short Form Health Survey Headache Specific 36 and 12 (SF-36 and SF-12), European Quality of Life 5 Dimensions (EQ-5D), MIGraine attacks- Subjective COGnitive impairements scale (MIG-SCOG), and The Migraine Work and Productivity Loss Questionnaire (MWPLQ).

### Measurement of migraine morbidity with patient reported outcome measures (PROMs)

Doctors use Patient Reported Outcome Measures (PROMs) as a means of measuring the impact of headaches on a patient’s daily function. This review summarises the currently available PROMs and their limitations. We contrast this with what headache patients value in terms of therapeutic success, on the basis of qualitative surveys, focus groups and interviews. Please see Table 1 for a summary of twenty of the currently available PROMs for migraine. Table 2 shows individual domains assessed by various PROMs which may impact patients during an attack. As Table 1 shows, only seven of the 20 PROMs includes content which have been co-developed with patients. Since the management of migraine has changed over time and there are no biomarkers to objectively tell us that treatment is effective, the gold standard barometer of treatment success should be the patient experience. However, a systematic review of headache PROMs found a significant lack of patient involvement in their creation which may mean a lack of relevance to what the patient is experiencing [8].

**Table 1:** Summary of patient reported outcome measures (PROMs) in migraine

PROM Name	Reference	Objective measures tested by the PROM	Response options	Recall period	Patient involvement:
HDI (Headache Disability Inventory)	Jacobson et al. 1994 [27]	25 items of functional and emotional impact	Mild, moderate, severe scoring system	Nil specified	No
HDQ (Headache-Specific Disability Questionnaire)	Niere et al. 2009 [28]	9 items of pain severity and functional impact	Scale of 0–10 (never/none to always/unable to do task)	The last month	No
HIT-6 (Headache Impact Test-6)	Kosinski et al. 2003 [29]	6 items about functional impact	Scale from never, rarely, sometimes, very often to always	The last 4 weeks	No
MIDAS (Migraine Disability Assessment)	Stewart et al. 1999 [30]	5 items about functional impact on chores, work and leisure	Number of days affected	The last 3 months	No
MSQv2.1	Martin et al. 2000 [31]	14 items about restrictions to work and leisure, prevention of work and leisure, and emotional impact	Number of days affected	The last 4 weeks	Yes
MSQOL (Migraine-Specific Quality of Life)	McKenna et al. 1998 [32]	14 items about functional impact on work, social life, energy levels, headache symptoms and general worries/concerns	6 point Likert scale from never, rarely, sometimes, pretty, almost, always	The last 4 weeks	Yes
HANA (Headache Needs Assessment)	Cramer et al. 2001 [33]	7 items about functional impact and mood	5 point Likert scale from never, rarely, sometimes, often to all the time	The last 4 weeks	Yes
Eurolight	Andree et al. 2010 [34]	103 items about headache disability, disease management and quality of life	7% open questions, 15% numerical responses (number needed), 78% categorical (tick or no tick)	Varies from “yesterday”, the last 30 days, last 3 months, “the last year” and “last day you had headache”	No
CORS (Completeness of response to migraine therapy)	Coon et al. 2012 [35]	32 items related to satisfaction of migraine therapy e.g. completeness of relief, speed of relief, return to functionality, fatigue, overall satisfaction etc.	5 point Likert scale of zero/none of the time to four/always	Response to single dose of medication and its effect within 2 h and 24 h respectively	Yes
Migraine-ACT (migraine assessment of current therapy)	Dowson et al. 2004 [36]	4 items for evaluation of acute medication e.g. speed, extent and consistency of relief	Yes/no responses to all 4 questions	Last 2 h since taking acute medication	No
MTAQ (Migraine Therapy Assessment questionnaire)	Chatterton et al. 2002 [37]	9 items to identify potential management issues e.g. migraine control, treatment satisfaction and economic burden of treatment	Yes/no responses to all questions	Last 2 h since taking acute medication	Yes
M-TOQ-5 (Migraine Treatment Optimisation-5 questionnaire)	Lipton et al. 2009 [31]	5 items about treatment optimisation	Yes/no responses	Varies from last 2 h of taking acute medication to last 24 h	No
M-TOQ-15 (Migraine Treatment Optimisation-15 questionnaire)	Lipton et al. 2009 [38]	15 items about treatment optimisation	Yes/no responses	Varies from last 2 h of taking acute medication to last 24 h, up to last 4 weeks	No
M-TOQ (Migraine Treatment Optimisation questionnaire)	Lipton et al. 2009 [38]	19 items about treatment optimisation	Yes/no responses	Varies from last 2 h of taking acute medication to last 24 h, up to last 4 weeks	No
PPMQ-R (Patient perception of migraine treatment- revised)	Revicki et al. 2006 [39]	29 items about satisfaction with migraine treatment e.g. efficacy, function, cost, side effects	7 point Likert scale from very satisfied to dissatisfied	Varies from last 24 h to last 4 weeks	Yes
SF-36 (Short Form Healthy Survey 36)– headache specific	Magnusson et al. 2012 [40]	36 items about quality of life and disability with general questions about health, physical health, body pain, and general pain	5 point Likert scale from poor-excellent, 5 point Likert scale from much better to much worse, 3 point scale from limited a lot to limited a little, to yes/no answers	Last 4 weeks	No
SF-12 (Short Form Healthy Survey 12)	Ware et al. 1996 [41]	Quality of life and disability	5 point Likert scale from poor-excellent, 5 point Likerty scale from much better to much worse, 3 point scale from limited a lot to limited a little, to yes/no answers	Last 24 h to last week	No
EQ-5D (European Quality of Life 5 Dimensions)	EuroQOL Group 1990 [42]	15 items about quality of life	Tick for yes/no	Today	No
MIGraine attacks- Subjective COGnitive impairments scale (MIG-SCOG)	Gil Gouveia et al. 2011 [43]	9 items about subjective cognitive complaints regarding language and executive function	3 possible responses: often/sometimes/no	Not specified	Yes
The Migraine Work and Productivity Loss Questionnaire (MWPLQ)	Lerner et al. 1999 [44]	29 items to assess impact of migraine and its therapies on paid work	6 point Lickert scale ranging from ‘no difficulty’ to ‘couldn’t do at all’	Most recent episode of migraine	Yes

**Table 2:** Domains assessed by patient reported outcome measures (PROMs) in migraine

PROM Name	Headache pain characteristics e.g. frequency, intensity or duration	Associated headache features e.g. nausea, photophobia	Impact on Household tasks or chores	Impact on Education or Employment	Impact on social life or leisure	Impact on relationships or family	Impact on psychological function e.g. mood, anxiety or depression	Impact on cognition and thinking skills	Treatment impact e.g. access or satisfaction with Rx
HDI (Headache Disability Inventory)	Yes	No	Yes	Yes	Yes	Yes	Yes	Yes	No
HDQ (Headache-Specific Disability Questionnaire)	Yes	No	Yes	Yes	Yes	Yes	No	No	No
HIT-6 (Headache Impact Test-6)	Yes	No	Yes	Yes	Yes	No	Yes	No	No
MIDAS (Migraine Disability Assessment)	No	No	Yes	Yes	Yes	No	No	No	No
MSQv2.1	No	No	Yes	Yes	Yes	Yes	Yes	No	No
MSQOL (Migraine-Specific Quality of Life)	No	No	Yes	Yes	Yes	Yes	Yes	No	No
HANA (Headache Needs Assessment)	No	No	No	No	Yes	Yes	Yes	Yes	No
Eurolight	Yes	Yes	Yes	Yes	Yes	Yes	Yes	No	Yes
CORS (Completeness of response to migraine therapy)	No	Yes	No	No	No	No	Yes	Yes	Yes
Migraine-ACT (migraine assessment of current therapy)	No	No	No	No	No	No	No	No	Yes
MTAQ (Migraine Therapy Assessment questionnaire)	Yes	No	No	Yes	Yes	No	No	No	Yes
M-TOQ-5 (Migraine Treatment Optimisation-5 questionnaire)	No	No	Yes	Yes	Yes	Yes	No	No	Yes
M-TOQ-15 (Migraine Treatment Optimisation-15 questionnaire)	Yes	Yes	Yes	Yes	Yes	Yes	Yes	No	Yes
M-TOQ (Migraine Treatment Optimisation questionnaire)	Yes	Yes	Yes	Yes	Yes	Yes	Yes	No	Yes
PPMQ-R (Patient perception of migraine treatment- revised)	No	Yes	No	No	Yes	Yes	Yes	Yes	Yes
SF-36 (Short Form Healthy Survey 36)– headache specific	Yes	No	Yes	Yes	Yes	Yes	Yes	No	No
SF-12 (Short Form Healthy Survey 12)	No	No	Yes	Yes	Yes	Yes	Yes	No	No
EQ-5D (European Quality of Life 5 Dimensions)	Yes	No	Yes	Yes	Yes	Yes	No	Yes	No
MIGraine attacks- Subjective COGnitive impairments scale (MIG-SCOG)	No	No	No	No	No	No	No	Yes	No
The Migraine Work and Productivity Loss Questionnaire (MWPLQ)	No	No	Yes	Yes	No	No	Yes	Yes	Yes

Table 1 shows that most PROMs look at quality of life and reduced function in general terms (HDI, HDQ, HIT-6, MIDAS, MSQv2.1, MSQOL, HANA, Eurolight, SF-36, SF-12, Eq. 5D), while specific scales are used to measure efficacy of acute migraine treatment (CORS, migraine-ACT, MTAQ, MTOQ, MTOQ-5, MTOQ-15, PPMQ-R). To fill gaps in many of these existing migraine disability scoring systems, the MIG-SCOG and MQPLQ were developed to quantify the burden of cognitive impact and productivity in paid work due to migraine respectively. Each of these scoring systems differs with respect to content, response format and recall period. There is also a considerable difference between PROMs with respect to length; the Eurolight has 103 questions which makes it incredibly comprehensive but could be a deterrent to patients due to the time requirement and need for sustained attention for complete all the items. In contrast, the Migraine-ACT only has four questions which makes it quick to administer, but possibly at the detriment of achieving a complete picture of the patient’s experience.

As Table 2 shows, the various PROMs assess the following domains with their specific questions: headache pain characteristics 9/20 (45%), headache associated features 5/20 (25%), impact on household tasks 14/20 (70%), impact on education or employment 15/20 (75%), impact on social life 16/20 (80%), impact on relationships or family 13/20 (65%), impact on cognition 7/20 (35%), impact on mood 13/20 (65%) and efficacy/satisfaction with migraine treatment 9/20 (45%). No PROM assessed all these domains. Lack of coverage of all possible symptoms that can affect quality of life is not necessarily a shortcoming, however, as a specific scale can be used to track symptoms of interest, e.g. MIG-SCOG for a patient affected with predominant brain fog due to migraine.

While there are many PROMs available for evaluating headache-related morbidity, it is interesting that the language used may not be comprehensible to most patients [9]. A study by Hazewinkel et al. [9] found that despite the recommended reading level for patient-facing material being grade six or lower, none of the currently available PROMs met this standard. They found that 14% of PROMs were grade seven or eight reading level, and the remaining 86% of PROMs were beyond grade eight level. The PPMQ-R was at a grade 13 reading level. This is significant when studies have shown that 20% of American adults cannot even understand grade-four reading level [9]. The implication of this is that the currently available PROMs likely include items which cannot be understood by the average patient thus diluting the likelihood that we are capturing morbidity data that is accurate and reflective of the true patient experience. In turn, if we cannot understand the current state of play, it is likely that the metrics of patient-reported treatment success are also inaccurate. One could also speculate that in the longer term, patients may be less likely to contribute longitudinal data on these PROMs, which further hampers our ability to track treatment successes or failures over time. In addition to all these concerns, from an ethical standpoint, if only patients of certain education levels can understand the PROMs then our understanding of migraine is greatly underrepresenting the experiences of minority or under-privileged groups.

In a similar vein, a study looking specifically at the patient experience using the HIT-6 migraine disability scoring system, found many areas to improve upon [10]. Nine headache patients were asked to complete the HIT-6 whilst reading aloud the questions and commenting on their thoughts about what the question meant, how easy the question was to understand and any confusion in interpretation of content. Similar themes emerged. Patients were unsure whether the disability questions related to function before or after acute medications were taken. Patients had difficulty with statements that contained contradictory examples to their eye such as ‘In the past four weeks, how often have headaches interfered with your leisure time activities such as reading or exercising?’ Several participants commented that exercise was not a leisure activity, so were unsure how to respond. The wording of responses also created some confusion because ‘always’ vs. ‘never’ were understood more easily than ‘definitely true’ vs. ‘definitely false’. Qualitative analysis of existing PROMs thus offers an insight into how patients view the content and thus how their answers are generated.

The way in which PROMs should be designed has been previously well described [11]. We must first work out what is important to the patient, and then the best way to measure it. The best way to figure out what to measure relates to the patient experience through focus groups or qualitative interviews. They describe that measuring the outcome of interest e.g. reduction in pain, can be done via three ways: endpoints, measures and scores. Endpoints are whether a certain percentage of patients in treatment group will be pain free. Measures include looking at headache diaries for trends before and after therapy. Scores relate to pain scores out of 10, for example. The International Headache Society has recommendations on what measures of treatment efficacy could be used, and these differ between episodic and chronic migraine [11]. For example, episodic migraine focus more on efficacy of abortive therapies within two hours of taking a dose or relief of their most bothersome symptom; whereas chronic migraine patients have efficacy of their preventers judged by the reduction in monthly migraine days or changes to their overall functional status.

### Patient-led development of headache-related proms

In an effort to understand the patient experience and create relevant PROMs, 77 patients diagnosed with migraine were qualitatively interviewed to understand the patient experience [12]. At the conclusion of the interviews, the responses led to identification of 66 concepts which could be broken down into 12 for physical impact, 16 for cognitive impact, 10 for social impact, 19 for psychological impact and nine for treatment impact. The qualitative descriptions of how patients are affected are included in the paper. The authors recommend using these themes self-identified by patients in future PROMs, to ensure relevance to the target population. Overall, this work showed that the existing PROMs do include many of these themes but could include more inclusive language; questions asking about migraine impairing ability to care for family exclude patients who may have carer roles looking after neighbours, friends or pets.

So which symptoms do patients actually want resolved? Most clinical trials use changes to the three cardinal symptoms of migraine, namely nausea, photophobia and phonophobia, as surrogates of acute medication success [13]. Of 6045 patients with migraine who were surveyed about their most bothersome symptoms, 49% (2967/6045) reported photophobia as their most bothersome symptom; 28% (1697/6045) reported nausea and 23% (1381/6045) reported phonophobia [13]. Almost 65% reported that all three cardinal symptoms were present. Use of this as an endpoint or PROM would allow us to target therapeutics to responses that the patient actually wishes to resolve based on what interferes with their quality of life.

A suggestion to creating a more patient-centric outcome measure is to calculate morbidity at an individual level, rather than a societal level [14]. In this study, authors propose that we switch from calculating ‘years lived with disability’ to ‘time lost due to an attack (TLA)’– the latter of which is duration of a migraine attack multiplied by the degree of functional impairment, in order to quantify the individual impact of a migraine. This switches the focus from a population-level back to the individual patient. By calculating TLA at various time points within an attack, recovery can also be tracked. The authors also posit that patients may find it easier to quantify function, which is a positive concept, more readily than disability, which is a negative concept. Validation studies are, of course, required before application of this tool on a large scale but shows alternative ways of quantifying burden of migraine for individual patients which allows them to take into account prodromal symptoms, the aura, the headache and even the postdrome.

A group in Switzerland recently attempted to involve migraine patients in decision-making about meaningful changes to their treatment to develop a migraine-disability rating scale [15]. Ten migraine patients were interviewed to work out common themes of satisfaction with migraine treatment; their responses were collated and presented to a further 300 migraine patients for feedback as to whether this was reflective of the treatment wishes of the cohort as a whole. From an initial 200 items, the top 18 items were incorporated into a Functional Assessment of Migraine Scale-Research (FAMS-R). The responses were to be rated on a 5 point Likert scale from strongly disagree to strongly agree. While this group showed that creation of such a scale was possible, they did not run a pilot study to test their creation so it is difficult to know whether it has real world application.

### The use of proms for evaluating efficacy of acute medications in migraine

When designing PROMs, we need to ensure that the targets for medications are in line with patient wishes. A study was conducted whereby 150 males and 150 females were chosen from a migraine database to take part in creation of a headache PROM [16]. Patients were asked two open-ended questions, “If a new medicine was developed for migraine attacks, what would you wish the effect of this medication to be?” and “What do you find most bothersome about having a migraine attack?” The answers from the first round were then presented to the same patients in two further rounds, where patients were asked to rank the original responses to evaluate the desirability of various therapeutic outcomes. In contrast to the existing PROMs that deem success of a medication as relieving headaches, photophobia, phonophobia and nausea within two hours of medication ingestion, patient wishes were quite different. Patients wanted the abortive medication to take effect within 30 min, prevent worsening of pain after taking medication, to ensure they could function within one hour of medication, and to prevent recurrence of symptoms on the same day. This disconnect between patient and doctor metrics of therapeutic success show the benefit of including patients in creation of PROMs.

A study of over 1500 patients randomised to rizatriptan or placebo for moderate-severe migraine found that faster pain relief was associated with more satisfaction with migraine treatment [17]. In this study, patients were assessed at baseline and two hours after taking the medication or placebo; 60–70% of patients with mild pain after two hours reported some satisfaction with treatment but this percentage reduced with higher baseline pain, longer time to achieve pain reduction and presence of associated symptoms. This study has shown that rapid pain relief is a key determining factor in patient satisfaction with therapy, and confirms that this should be a key outcome measure in studies or trials involving acute medications for migraine.

A systematic review sought to evaluate the endpoints used in clinical trials [18]. Broadly, all the acute clinical trials had 4 types of outcomes measured– pain-related outcomes such as pain relief and extent of pain relief; presence of associated disabling symptoms such as photophobia or nausea; extent of disability or impairment; and patient-reported outcome measures. They found a great degree of heterogeneity in what the outcome measures were, how they were measured and the timing of outcome measurements, e.g. from 10 min to 24 h post treatment. The endpoints varied from a specific time point (e.g. three months from start of study), responder definition (success of treatment being at least 50% reduction in headaches) and changes from baseline (e.g. changes in MIDAS scores). Having more consistency in reporting, with patient input, would allow for meaningful changes in treatment to be captured and allow comparison between trials. The currently assessed end points of trials include pain relief at two hours (i.e. reduction in pain intensity from moderate-severe down to mild-no pain), pain freedom at two hours (no pain at all), sustained pain freedom for 24 h (with no need for further acute medications within this time), and sustained pain freedom with no adverse effects (hardest to achieve since it combines all the other previous markers as well) [19]. These endpoints may not necessarily be reflective of what patients are after with their treatments– as we have previously touched on, patients would ideally want pain relief within 30 min when polled and to be functional within one hour [16].

### The use of proms for evaluating efficacy of preventer medications in migraine

A study aimed to look at the gold standard PROM which could be used to assess efficacy of migraine preventers at 12 weeks with specific mention of the anti-CGRP therapy [20]. As the International Headache Society guidelines look at monthly migraine days as a marker of migraine severity, end points for preventer trials often look at reductions in monthly headache days as end points. Seven PROMs were given to patients before and after starting treatment. Only the MSQ showed treatment efficacy across all questions, and the MIG-SCOG was the only PROM to not show any differences before and after treatment. Specifically an improvement in MSQ scores of 18% or more correlated with a decision to continue with anti-CGRP therapy [20]. This study shows the benefit of marrying up a PROM with treatment decision-making to guide future therapy. The scales can also help us to find a treatment efficacy scales that guide ongoing treatment which are meaningful to patients, rather than relying purely on reduction in monthly migraine days or days of acute medication intake.

This was a good study to not just issue patients with PROMs to fill out to check their general quality of life, but a tool which allows us to see if there is a meaningful change to therapy.

### New approaches to the development of headache-specific outcome measures

A recent literature review and meta-analysis of headache-related disability in over 130,000 patients with primary headache disorders found common headache PROMs do not capture the full patient experience of headache disorders [21]. In particular, many PROMs fail to capture the interictal burden, work-related productivity losses, absenteeism and economic impacts [21]. They do, however, comment that different PROMs have different advantages such as the HIT-6 capturing mental impacts of headache which the MIDAS fails to do since it focuses more on a patient’s role within the family and work domains [21]. Perhaps since migraine is a chronic condition with potentially years of specialist follow up, these different PROMs could be administered at different time points based on a patient’s priority at the time to ensure all facets of a patient’s life are optimised. This review reiterates the difficulty in creating one single PROM that is all-encompassing when considering that primary headaches often co-exist with other pain disorders, anxiety and depression which all increase morbidity and thus makes a real-world headache PROM difficult to assess without significant confounders [21].

As demonstrated so far in our review, there is a paucity of patient-involvement in the development of headache PROMS. Models of consumer satisfaction often talk about bridging the ‘gap’ between what a patient expects and what they perceive that they are receiving [22]. Accordingly, bridging this gap should increase satisfaction with migraine therapy. The Migraine Treatment Satisfaction Measure (MTSM) was created as a means of working out which aspects of acute treatment were important to patients [22]. Nine items were conceived by physicians and then presented to 29 migraine patients before and after starting any new acute pain medication: pain relief, speed of pain relief, freedom from pain, additional symptoms, confidence in treatment, disruption in life, dosing, freedom from relapse, and ease of use. Patients had to rank these nine attributes from most to least important. This list was compiled from a physicians’ understanding of common complaints of migraine patients when starting new migraine medications. With this list, the format for a qualitative interview was developed and then presented to 54 patients in focus groups and individual patient interviews conducted to compile items for the MTSM. The thinking behind this is that a global score of 30, for example, may not mean that several patients with the same score are equally satisfied. A patient who places a very high importance to speed of pain relief whose pain is relieved faster than expected may have a score of 30 and be very happy. Another may place speed of relief as lower in importance but achieve faster relief than expected so have a score of 30. They may not have placed the same importance on different qualities thus may have different satisfaction levels. The MTSM is undergoing ongoing studies in wider populations to determine its applicability to other population. Such approaches have been used in infectious diseases and within epilepsy cohorts to develop desirability of outcome ranking measures as shown below.

A potential way to incorporate the patient experience, as well as outcomes measures traditionally used by physicians, is to allow patients to rank desirable outcomes from most important to least important balancing potential benefits and harms of the treatment, such as has been done in antibiotics trials [23, 24] and epilepsy trials [25]. In the original development of such a ranking system for efficacy of antibiotics, there were five ordinal outcomes reported: clinical benefit without adverse effects of medications, clinical benefit with some adverse effects, survival without clinical benefits or adverse effects, survival without clinical benefit but with adverse effects, or death [23]. Higher rankings are more desirable for patients than lower rankings. Allowing sub-responses within each of these strata allow for more detailed ranking of outcomes that considers benefits and harms within the same patient as a composite outcome that can also be ordered on a quantitative level. In a similar vein, such a ranking system of outcomes in epilepsy patients has led to clinicians moving away from merely seizure freedom as a marker of successful epilepsy treatment and considers medication side effects, costs of medications and fear of future seizures as markers of treatment efficacy [25]. Due to high patient satisfaction, the patient-focused desirability of ranking is used as a primary outcome in clinical trials [26].

We propose that a migraine-specific DOOR could be created with consumer involvement. Taking chronic migraine as an example, we would first need to identify patients with this diagnosis who are happy to take part in focus groups. The focus groups would need to occur on an iterative basis whereby initial meetings would ask very open-ended questions which would become successively more refined and specific with time. Initial questions could include: What is your main priority when taking a migraine medication? What would be the qualities of an ideal migraine medication for you? What side effects do you wish to avoid from a migraine medication? What do you wish your headache doctor focused on more when picking a migraine medication for you? Once a list of management priorities from patients has been accumulated, this could be compared to traditional markers of treatment success such as reduction in migraine days [11], or relief of most bothersome symptoms [13]. The groups as a whole, rather than relying on individual patient opinions, would then be involved in ranking the outcomes in order to create the final ranked list of outcomes, using similar methodology to the epilepsy trials [25]. The epilepsy DOOR incorporates seizure reduction, avoidance of medication adverse effects and improved quality of life as markers of treatment success; a headache DOOR could look similar by having a composite outcome measure of reduction in migraine days, avoidance of medication side effects and improvement of quality of life. To this end, successive focus groups should involve different patients to the original focus groups so that we get a wider cross-sectional opinion on patient preferences. Allowing for consumer engagement with comments, feedback and suggestions through social media or migraine advocacy organisations may also allow for a larger subset of the population to have an input into a migraine DOOR. We also advocate for separate DOORs for episodic-migraine and chronic-migraine since the phenotype varies, and so too should our treatment approaches.

How a migraine DOOR is received by regulatory authorities or physicians managing this condition is unknown. However, given its potential to increase patient satisfaction in a more meaningful way and therefore potentially reduce hospital presentations or even days off work, its validation through prospective clinical trials in headache may pave the way for its wider implementation.

### Future directions

Given the significant lack of patient involvement in most existing headache-PROMs, there is a lack of understanding about whether current care mirrors patient expectations. Recent introduction of the consumer informed DOORs incorporating assessments of benefits and harms of the treatment, as an alternative to traditional PROMs, may be a novel approach to enhancing patient-centred care.

## Data Availability

No datasets were generated or analysed during the current study.

## Abbreviations

CORS: Completeness of response to migraine therapy
EQ-5D: European Quality of Life 5 Dimensions
HANA: Headache Needs Assessment
HDI: Headache Disability Inventory
HDQ: Headache-Specific Disability Questionnaire
HIT6: Headache Impact Test
MIDAS: The Migraine Disability Assessment Test
Migraine-ACT: Migraine assessment of current therapy
MIG-SCOG: MIGraine attacks- Subjective COGnitive impairments scale
MSQ: Migraine Specific Quality of Life Questionnaire
MSQOL: Migraine-Specific Quality of Life
MTAQ: Migraine Therapy Assessment questionnaire
M-TOQ-5: Migraine Treatment Optimisation-5 questionnaire
M-TOQ-15: Migraine Treatment Optimisation-15 questionnaire
M-TOQ: Migraine Treatment Optimisation questionnaire
MWPLQ: The Migraine Work and Productivity Loss Questionnaire
PPMQ-R: Patient perception of migraine treatment- revised
PROMS: Patient reported outcome measures
SF-12: Short Form Healthy Survey 12
SF-36: Short Form Healthy Survey 36
